# Anxiety, Depression and Quality of Life among youth in Kosovo

**DOI:** 10.1192/j.eurpsy.2025.1474

**Published:** 2025-08-26

**Authors:** N. Fanaj, S. Mustafa, E. Krasniqi

**Affiliations:** 1 Alma Mater Europae Campus College Rezonanca, Prishtina; 2 PMSH, Prizren; 3 AAB College; 4 UBT College, Prishtina, Kosovo

## Abstract

**Introduction:**

The literature review on anxiety/depression rates and quality of life (QoL) in youth in Kosovo are scarce. Studies on anxiety and depressive symptoms in Kosovo mostly, have revealed high prevalence rates pre and during COVID-19.

**Objectives:**

This study aimed to explore the relationship between anxiety, depression, and quality of life in Kosovo youth. The significance of this study lies in the fact that these variables are being examined together for the first time in Kosovo after COVID-19.

**Methods:**

Its cross-sectional study. The sample consisted of 563 students aged 18 to 25 (Mage=20.88; SD=1.52). Participants recruited online in the period of May 2023, and completed the Albanian version of GAD-7, PHQ-9 and GQOL (Global Quality of Life Scale). Data processing was done with SPSS 27.0 and Microsoft Excel 2019.

**Results:**

The prevalence of anxiety (measured with GAD-7, ≥10) was 42.6 % while the prevalence of depression (measured with PHQ-9, ≥10) was 45.5%. Significant negative correlations were observed between anxiety and QoL (r = -.335, p < 0.00) and between depression and QoL (r = -.326, p < 0.00). A partial correlation indicated that anxiety and depression had very much influence in controlling for the relationship with QoL reciprocally; for anxiety (r = -.133, p < 0.02) and for depression (r = -.109, p < 0.13). Females and low SES emerged (but not employment status) as factors associated with higher levels of anxiety/depression and low Quality of Life.

**Conclusions:**

Our anxiety/depression prevalence rates are mostly above those found in Kosovar youth both before and during COVID-19, and also in comparison to other European countries. Anxiety and depression, both separately and especially when co-occurring, significantly affect the quality of life of young people. Despite the fact that prevalence rates can vary depending on the specific methodology, sample size, and population demographics of each study, these findings should be taken seriously and addressed by mental health authorities in Kosovo through preventive and treatment programs. Additionally, further studies with sound scientific quality are necessary.

**Disclosure of Interest:**

None Declared

